# A Belated Green Revolution for *Cannabis*: Virtual Genetic Resources to Fast-Track Cultivar Development

**DOI:** 10.3389/fpls.2016.01113

**Published:** 2016-07-29

**Authors:** Matthew T. Welling, Tim Shapter, Terry J. Rose, Lei Liu, Rhia Stanger, Graham J. King

**Affiliations:** ^1^Southern Cross Plant Science, Southern Cross UniversityLismore, NSW, Australia; ^2^Ecofibre Industries Operations Pty LtdMaleny, QLD, Australia

**Keywords:** genetic conservation, industrial hemp, marijuana, phenotypic variation, plant breeding, cannabinoids

## Abstract

*Cannabis* is a predominantly diecious phenotypically diverse domesticated genus with few if any extant natural populations. International narcotics conventions and associated legislation have constrained the establishment, characterization, and use of *Cannabis* genetic resource collections. This has resulted in the underutilization of genepool variability in cultivar development and has limited the inclusion of secondary genepools associated with genetic improvement strategies of the Green Revolution. The structured screening of *ex situ* germplasm and the exploitation of locally-adapted intraspecific traits is expected to facilitate the genetic improvement of *Cannabis*. However, limited attempts have been made to establish the full extent of genetic resources available for pre-breeding. We present a thorough critical review of *Cannabis ex situ* genetic resources, and discuss recommendations for conservation, pre-breeding characterization, and genetic analysis that will underpin future cultivar development. We consider East Asian germplasm to be a priority for conservation based on the prolonged historical cultivation of *Cannabis* in this region over a range of latitudes, along with the apparent high levels of genetic diversity and relatively low representation in published genetic resource collections. Seed cryopreservation could improve conservation by reducing hybridization and genetic drift that may occur during *Cannabis* germplasm regeneration. Given the unique legal status of *Cannabis*, we propose the establishment of a global virtual core collection based on the collation of consistent and comprehensive provenance meta-data and the adoption of high-throughput DNA sequencing technologies. This would enable representative core collections to be used for systematic phenotyping, and so underpin breeding strategies for the genetic improvement of *Cannabis*.

## Introduction

*Cannabis*, an erect annual herb and member of the *Cannabaceae* family, is monotypic and characterized by a single species *Cannabis sativa* L. (Small and Cronquist, 1976). Plants are diploid (2*n* = 20) with an estimated haploid genome of ~830 Mb (Van Bakel et al., 2011). The extant genepool is thought to be comprised primarily of domesticated or feral populations, cultivars, and selections (Small and Cronquist, 1976), with a subset having been subject to steep selective gradients toward phenotypes for specific end-uses (Mandolino and Carboni, 2004; Potter, 2014; Small, 2015a). *Cannabis* has been cultivated in Eurasia over several thousand years (Li, 1974; Bradshaw et al., 1981; Murphy et al., 2011; Herbig and Sirocko, 2013) and has since radiated from this region and been subject to prolonged artificial selective pressures in Africa (Duvall, 2016) and North and South America (Small and Marcus, 2002), and is now cultivated globally (Salentijn et al., 2014). Plants are diecious and obligate outbred, although some fiber forms are monecious (Faeti et al., 1996). This has contributed to a high level of hybridization between pre-, post-, and de-domesticated populations (Gilmore et al., 2007), and therefore few if any intact wild populations are thought to exist (Small and Cronquist, 1976).

The Green Revolution saw the productivity of major crops such as wheat (*Triticum* spp.), rice (*Oryza sativa*), maize (*Zea mays*), sorghum (*Sorghum bicolor*), and millet (*Pennisetum glaucum*) increase by 0.5–1% annually between 1960 and 2000 (Evenson and Gollin, 2003). A large proportion of these gains has been attributed to germplasm improvements initiated by the Consultative Group on International Agricultural Research (CGIAR; Hajjar and Hodgkin, 2007), which facilitated genetic resource exchange, adaptive transfers, and the free flow of materials and knowledge between research groups (Pingali, 2012). However, the genetic improvement of crop-types was not evenly distributed (Evenson and Gollin, 2003). During the latter part of the twentieth century legitimate crop-types of *Cannabis*, commonly referred to as industrial hemp, not only failed to benefit from advances in breeding technologies and genetic resource utilization, but also suffered significant losses in *ex situ* conservation (Clarke, 1998; Small and Marcus, 2002; Ranalli, 2004), with likely significant genetic erosion, as has been documented with the loss of rice wild relatives in China (Gao, 2003).

The major contributing factor to the erosion of *Cannabis* genetic resources and limited genetic improvement of industrial hemp has been the status of *Cannabis* as the source of one of the most widely used illicit drugs (Hall and Degenhardt, 2007). Marijuana refers to an informal subtaxa of *Cannabis* which has been selected specifically for its psychoactivity and propensity to accumulate high levels of delta-9-tetrahydrocannabinol (THC; Hewavitharana et al., 2005). The prohibition on marijuana, which is morphologically indistinguishable from other forms of *Cannabis* (Small and Cronquist, 1976; Small, 2015a), has contributed to the decline of industrial hemp (Small and Marcus, 2003; Piluzza et al., 2013; Small, 2015a). In recent years there has been growing public acceptance that medical cannabis has therapeutic applications, which has been particularly prominent in a number of states of the USA, along with Australia, Canada, Israel, and Uruguay. This growing public tolerance toward *Cannabis* in North and South America (Schuermeyer et al., 2014) and in other parts of the world has led to a number of political and legal reforms in these legislatures (Pardo, 2014), and suggests not only a renewed consumer demand for *Cannabis*-related products, but also an increased requirement for breeders to develop varieties for specific end-use applications.

This deterioration in conservation of industrial hemp genetic resources is not consistent with the intrinsic functionality or agricultural value of the species. The long pericyclic and phloem fibers in the bast fraction of the culm (stem) have high tensile strength, and were used for millennia as the predominant source of fiber for the production of rope (Murphy et al., 2011). They have also been used in the textile and paper industries (Amaducci et al., 2014), as well as in fiber mat thermoplastics for automobile manufacture (Pervaiz and Sain, 2003). The woody core or hurd (shives) within the xylem vascular tissue, comprising up to 70% of the stem biomass (De Meijer, 1994), has also found minor low value use in the production of lime-hemp concrete, a light composite building material (De Bruijn et al., 2009), as well as animal bedding and garden mulch (Pervaiz and Sain, 2003). Achene (seeds) are also suitable for human and animal consumption and contain all essential amino acids (Callaway, 2004), with the exception of lysine, (House et al., 2010) in a readily bioavailable form (Malomo and Aluko, 2015), as well as a balanced omega-6/omega-3 essential polyunsaturated fatty acid (PUFA) ratio (Callaway, 2004; Carvalho et al., 2006; Da Porto et al., 2012).

In addition to the traditional uses in paper and textile industries, a number of novel end-use applications for *Cannabis* are currently being explored. These include cellulosic bioethanol which can be extracted from plant biomass and utilized as a potential transportation biofuel (Finnan and Styles, 2013; Kuglarz et al., 2016). Higher value products being assessed include cellulose nanocrystals, which have been generated from low quality fibers (Li et al., 2010) for the development of high-performance nanocomposites (George and Sabapathi, 2015), while graphitic carbon nanosheets developed from hemp bast fiber precursors have been shown to have ionic-liquid-based supercapacitor properties (Wang et al., 2013).

The phenolic compounds *N*-trans-caffeoyltyramine and cannabisin B (Chen et al., 2012), as well as prenylflavonoids cannflavins A and B (Werz et al., 2014) occurring within seed husks and sprouts, respectively, may have potential nutraceutical applications. In addition, *Cannabis* plants produce a group of pharmacologically active terpenophenolic phytocannabinoids (cannabinoids; ElSohly and Slade, 2005; Appendino et al., 2011). These compounds are currently fueling an emerging cannabinoid-based pharmaceutical industry (Potter, 2014), with botanical extracts being safely and efficiently administered via oromucosal sprays (Izzo et al., 2009) and by electrically-driven vaporizers (Lanz et al., 2016). Other terpenoid-related compounds that are present at high concentrations in *Cannabis* may also contribute to pharmacological entourage effects (Russo, 2011), or have their own specific medicinal applications, such as α-humulene which has antifungal properties potentially beneficial in the treatment of cryptococcosis (Wanas et al., 2016).

The traditional and still predominantly successful approach to crop genetic improvement has been based on exploiting naturally occurring genetic diversity. Exploitation of genetic diversity can be achieved through the hybridization of elite breeding material with exotic germplasm, defined here as a genetic resource which has not been artificially subject to a high level of selection for a given targeted trait or growing environment. In the case of heterozygote breeding lines, extensive trait diversity can be uncovered by crossing siblings of the same accession and observing transgressive trait segregation within the progeny. A recurrent introgressive population enrichment approach can then be used to generate elite populations and cultivars with improved performance or novel properties. However, this requires access to structured plant genetic resources (Babic et al., 2015; Scossa et al., 2015). *Ex situ* genetic resources provide a valuable pool of genetic diversity (Hajjar and Hodgkin, 2007) and have been proven to be an essential source of allelic variation for trait improvement (Hajjar and Hodgkin, 2007; Castañeda-Álvarez et al., 2015; Scossa et al., 2015). Wild or under-domesticated germplasm, in the form of either crop wild relatives or locally-adapted landraces, provide a repository to reintroduce allelic diversity into domesticated populations (Vincent et al., 2013).

**Figure 1:**
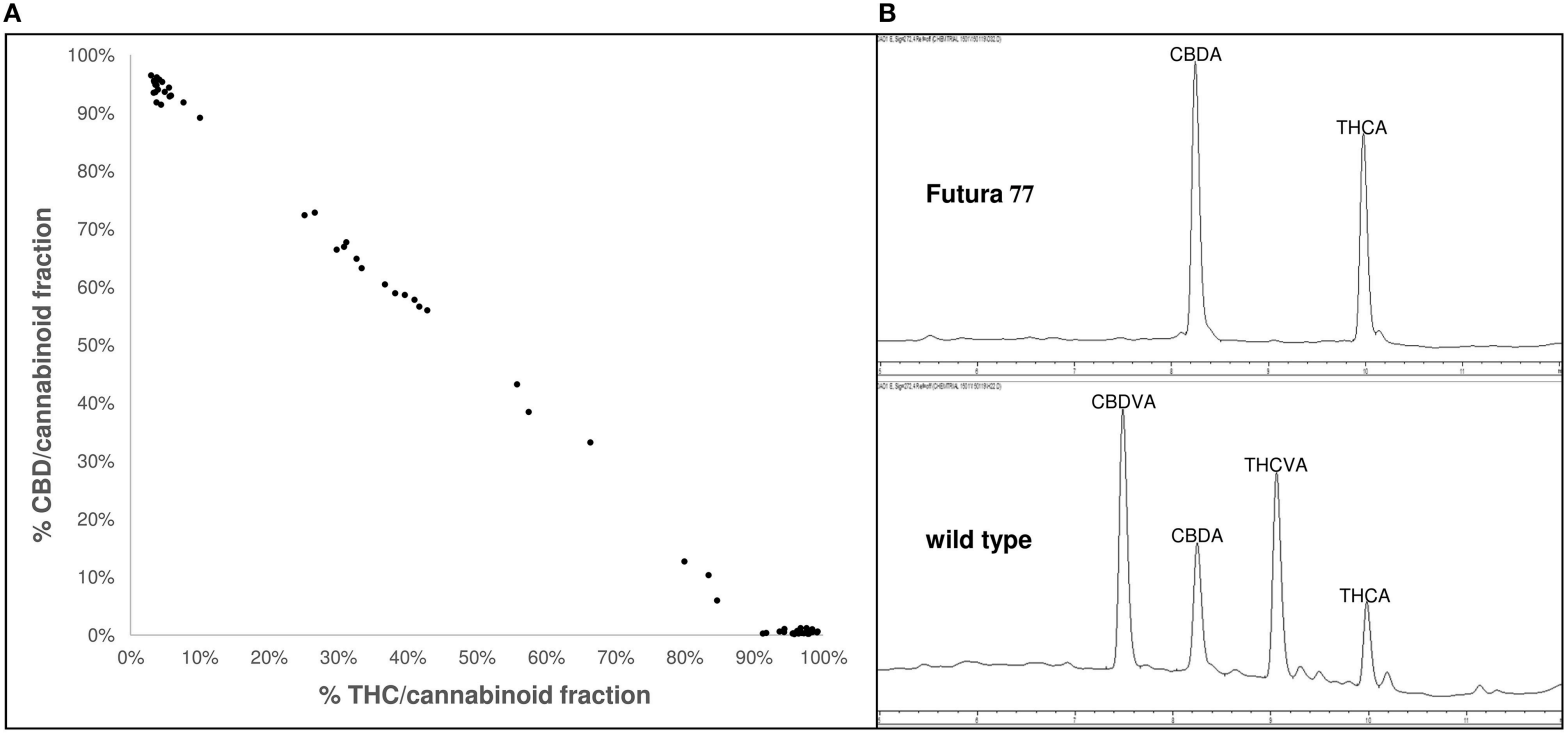
**Phenotypic variability in cannabinoid composition in exotic ***Cannabis*** germplasm (A) variability of acidic and neutral forms of the penty-alkyl-cannabinoids THC and CBD within the cannabinoid fraction**. *Data points* correspond to 66 plants derived from 22 accessions; Accessions were sourced from different geographical locations and represent different stages of domestication; Accessions were grown in environmentally-controlled conditions and harvested at similar developmental stages; redrawn from Welling et al. (2015); **(B)** chromatogram at 272 nm of a single cultivated and wild-type plant; *Top* chromatogram of an individual European cultivar Futura 77 exhibiting acidic forms of the pentyl-alkyl-cannabinoids CBD and THC as the predominant cannabinoids; *Bottom* chromatogram of an individual East Asian landrace exhibiting acidic forms of the propyl-alkyl-cannabinoids CBDV and THCV as the predominant cannabinoids; CBD, cannabidiol; CBDV, cannabidivarin; THC, delta-9-tetrahydrocannabinol; THCV, delta-9-tetrahydrocannabivarin.

**Figure 2:**
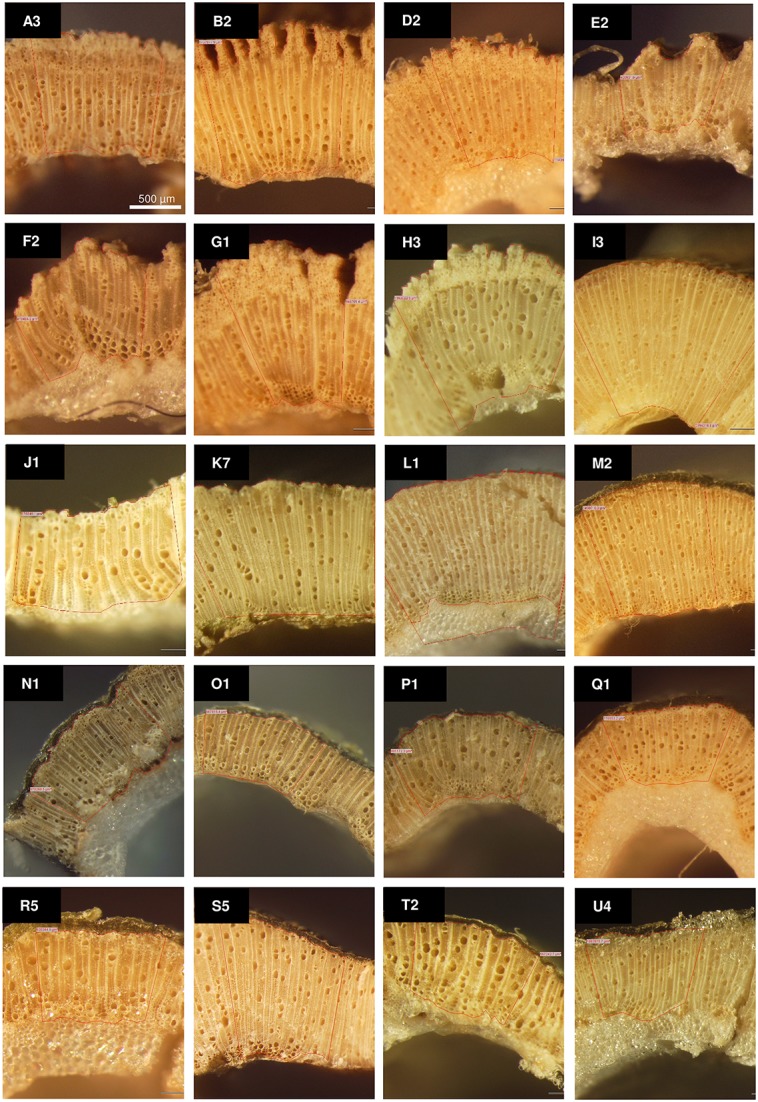
**Phenotypic variation in the xylem: phloem ratio for a range of ***Cannabis*** germplasm from the Ecofibre Global Germplasm Collection**. Stereo microscopy images taken from internode 3–4 across a representative sub-set of 20 accessions; Accessions were grown in environmentally-controlled conditions and harvested at similar developmental stages; *Letters* correspond to accessions and *numbers* to replicate plant; Scale bar: 500μm; redrawn from (Stanger, 2015).

Germplasm surveys of *Cannabis* have revealed substantial phenotypic diversity in oil content (Kriese et al., 2004; Matthäus et al., 2006; Grigor'ev et al., 2010), cannabinoid (Welling et al., 2015), and xylem composition (De Meijer, 1994). Exploitation of this phenotypic diversity could be used for the development of novel cultivars tailored for specific end-use applications. For instance, phenotypic diversity in the composition of THC and cannabidiol (CBD; Figure 1A) as well as other rare-alkyl-cannabinoid homologs (Figure 1B; Welling et al., 2015) has the potential to be used to develop breeding lines for the production of specialized and target-specific botanical drug products (Izzo et al., 2009). Phenotypic diversity in xylem composition (Figure 2) could also be exploited to increase xylem and hemicellulose quality to improve the suitability of *Cannabis* as a feedstock for the production of furfural (Brazdausks et al., 2016), a promising and versatile chemical precursor for a number of biofuels and bio-renewables, including plastics (see review López et al., 2016). However, at present the limiting step for introgression of exotic allelic variants into novel cultivars for these as well as other end-use and agronomic traits (Hillig, 2005a; Tang et al., 2013), is access to well-represented and characterized *Cannabis* genetic resources.

Explicit characterization of *Cannabis* genetic resources as a key stage in cultivar development has been underutilized (Mandolino and Carboni, 2004; Ranalli, 2004; Amaducci et al., 2014; Salentijn et al., 2014), and a more sophisticated use of intraspecific germplasm will be required to advance elite breeding lines able to meet market requirements and a wider range of growing environments worldwide. In this review we describe *Cannabis* genetic resources reported within the literature. Potential gaps in coverage of available *ex situ* collections, as well as recommendations for genetic resource management and the use of germplasm resources in crop improvement programs, are proposed. A novel virtual approach to *ex situ* conservation is outlined to facilitate the exploitation of *Cannabis* genetic resources within the legal constraints surrounding this plant.

## A hypervariable species shaped by domestication

### Taxonomy and classification

Patterns of genetic and phenotypic variation to inform prioritization of intraspecific groupings for *ex situ* conservation require an appreciation of current taxonomic nomenclature. *Cannabis* has traditionally been classified as monotypic, characterized by a single species *C. sativa* (Small and Cronquist, 1976). The presence of additional species has been inferred from allozyme variation (Hillig, 2005b) and chemotaxonomic analysis (Hillig and Mahlberg, 2004), and placed in context of Pleistocene climatic events which are believed to have resulted in the isolation and diversification of this genus (Clarke and Merlin, 2013). However, given the lack of validated reports of extant “natural” populations, one has to consider *C. sativa* as a species solely represented by domesticated or post-domesticated germplasm. Thus, claims of additional taxonomic divisions are controversial, on the basis that plants readily inter-cross and physiological and genetic barriers to gene flow appear absent (Small and Cronquist, 1976).

The use of allozyme variation and chemotaxonomic analysis to determine population structure tends to bias non-neutral selection. Moreover, allocating observed variation to specific loci and allelic status requires validation through segregation analysis. Electrophoretic variation in allozyme loci does not always correspond to DNA sequence variation, and may be associated with experimental artifacts (May and Hoelzel, 1992; Olsen et al., 2014), post-translational modification, *cis*-regulatory, or epigenetic processes (Olsen et al., 2014). Although chemotaxonomic differentiation has been inferred on the basis of cannabinoid composition (Hillig and Mahlberg, 2004), this is thought to be governed by only a handful of loci (De Meijer et al., 2009; Weiblen et al., 2015). Thus, the use of allozyme variation and chemotaxonomic analyses performed to date may provide limited information on the genetic composition of diverse accessions, and therefore lend little support for speciation.

Nonetheless, *Cannabis* is polymorphic (Small et al., 2003) and is often differentiated into intraspecific taxonomic groupings on the basis of chemotype, ecotype, crop-type (fiber or drug), and leaflet morphology (Small et al., 2003; Lynch et al., 2015). However, agreement on a practical and workable nomenclature for subspecies differentiation within *Cannabis* is lacking (Hillig, 2005b; Clarke and Merlin, 2015; Small, 2015b), and could be best described as *ad hoc*. The terms “sativa” and “indica” can be used variously to differentiate between recreational drug varieties, crop-types (Small and Cronquist, 1976), chemotypes (Elzinga et al., 2015), ecotypes, and changes in leaflet morphology (Hillig, 2005b; Clarke and Merlin, 2013). Classification based on the terms “fiber-type” and “drug-type” to differentiate between subspecies is also problematic, given the widespread changes in the status of *Cannabis* in different legislatures (Pardo, 2014) and the propensity for fiber accessions to exhibit characteristics indicative of both fiber- and drug-type plants (Tipparat et al., 2012; Welling et al., 2015). The use of descriptors associated with a combination of stable chemical and morphological traits, and/or that incorporate DNA evidence, is more likely to provide practical operational classification.

### Diversification through domestication

Anthropogenic selection focused predominantly on the traits defining industrial hemp, hempseed, pharmacological, and marijuana end-uses has been influential in the phenotypic and chemotypic diversification of *Cannabis* (see review Small, 2015a), and is therefore an important consideration for establishing the coverage and representation of germplasm accessions required for *ex situ* genetic resource conservation. Moreover, detection of population structure reflecting long-term crop use, post-domestication radiation, and eco-geographic adaptation may be valuable for the management of plant genetic resources and breeding programs, as has been demonstrated for the vegetable *Brassica oleracea* (Smith and King, 2000), a species which has been subject to similar patterns of renewed domestication into diverse crop forms and secondary radiation.

It has been suggested that domestication of marijuana has resulted in both qualitative and quantitative increases in THC. Analyses of THC content as a percentage of dried weight (w/w) as high as 39.8% have been recorded from contemporary marijuana preparations (Swift et al., 2013). However, such high levels in content could also be attributed to an improvement in analytical and extraction methodologies (De Backer et al., 2009) and the preferential sampling of cannabinoid-rich floral tissues (buds; Happyana et al., 2013), as opposed to demonstrable (specific) heritable genetic changes. Nonetheless, contemporary fiber and marijuana forms have shown that the latter can not only exhibit a higher overall cannabinoid content than the former, but also a higher proportion of THC within the cannabinoid fraction (Hillig and Mahlberg, 2004; Pacifico et al., 2006; Staginnus et al., 2014; Lynch et al., 2015). However, comprehensive large scale quantitative comparison both within and between representatives from each grouping are lacking.

Transcriptome analysis (Van Bakel et al., 2011; McKernan et al., 2015; Onofri et al., 2015; Weiblen et al., 2015) and QTL mapping (Weiblen et al., 2015) further support the historical selection pressure that has led to chemotypic separation between contemporary fiber and marijuana groupings. Cannabinoids accumulate in plants in their carboxylic acid forms, such as tetrahydrocannabinolic acid (THCA) and cannabidiolic acid (CBDA) which form neutral cannabinoids THC and CBD in a non-enzymatic reaction when exposed to heat (Dussy et al., 2005). The marijuana variety Purple Kush was found to have higher expression levels of genes encoding cannabinoid biosynthetic pathway intermediates than the fiber cultivar Finola, with Purple Kush only expressing functional sequence variants of genes coding for THCA and Finola those for CBDA synthase (Van Bakel et al., 2011). Linkage mapping in 62 F_2_ individuals derived from a cross between full-sib inbred contemporary fiber cultivar Carmen and marijuana variety Skunk#1 revealed QTL for THCA and CBDA composition, as well as putative QTL for cannabinoid content, with differences in composition associated with a CBDA synthase locus and loss of CBDA synthase functionality in Skunk#1 (Weiblen et al., 2015).

The occurrence of contemporary fiber cultivars which have relatively high levels of CBDA (Small, 2015a) can be attributed to breeding efforts within France and other European countries toward the middle to latter part of the twentieth century (Amaducci et al., 2014), where techniques such as the Bredemann method, an *in vivo* fiber evaluation method, and the counter selection for THC using marker assisted selection (MAS), were employed (Ranalli, 2004). Fifty one fiber cultivars are currently registered for use within the European Union (Salentijn et al., 2014) and these cultivars have been exported to North America (Small, 2015a) and the Northern provinces of China (Salentijn et al., 2014). Despite the scarcity of published data relating to ancestry of such accessions, it is believed that a large number of contemporary fiber cultivars are descendants from Central Russian and Mediterranean landraces and derivative cross-progenies (De Meijer and van Soest, 1992).

Phylogenetic relationships between domesticated *Cannabis* germplasm have recently been examined using reduced representation DNA sequencing. Genotyping by sequencing (GBS) analysis of 195 accessions using 2894 single nucleotide polymorphisms (SNPs) inferred close relatedness and shared ancestry between contemporary fiber accessions, with the latter forming a separate clade (Lynch et al., 2015). These accessions were also observed to exhibit lower levels of heterozygosity than other intraspecific taxa (Mann-Whitney U-test *p* < 0.001; Lynch et al., 2015), suggesting that recent domestication of fiber traits has resulted in a genetic bottleneck and reduction in allelic diversity. However, these varieties were not well-represented in the sample collection, with only 16 analyzed (Lynch et al., 2015). Moreover, a separate GBS study using 14,031 SNPs in 43 contemporary fiber and 81 marijuana varieties produced results that conflicted with this more recent study, and showed significantly lower levels of heterozygosity in marijuana varieties compared with fiber cultivars (Mann-Whitney U-test *p* = 8.64 × 10^−14^; Sawler et al., 2015).

Despite this lack of congruence between GBS analyses, domestication for either industrial hemp or marijuana traits has likely resulted in a loss of genetic and allelic diversity, potentially brought about by changes in breeding systems. Processes such as linkage drag can be associated with complex polygenic flowering QTL (Mace et al., 2013) in relation to latitudinal and environmental adaption (Gao et al., 2014). Regardless of reductions in allelic diversity that may have arisen either from clonal propagation in marijuana (Russo, 2007), or from the propagation of monoecious varieties in industrial hemp (Forapani et al., 2001), it is unclear to what extent contemporary *Cannabis* germplasm deviates from the broader genepool. Analysis of 45 SNPs in both GBS sample sets reveals an overall limited genetic distance between 22 industrial hemp and 173 marijuana groupings (Lynch et al., 2015). Resequencing and mapping of 30 billion sequence reads from 302 domesticated and wild soybean (*Glycine max*) accessions identified selective sweeps associated with domestication events (Zhou et al., 2015). By comparison, resequencing of various species of *Citrus* has also revealed a complex arrangement of large haplotype blocks and admixture between ancestral and domesticated species (Wu et al., 2014). On completion of a fully annotated *Cannabis* genome (Van Bakel et al., 2011), it may be possible to resequence diverse germplasm (Scossa et al., 2015) to quantify differences in genetic diversity and to determine the contribution wild ancestors have conferred to contemporary forms. However, access to wild and landrace accessions may be a limiting factor to exploring *Cannabis* phylogeny.

### Identifying centers of diversity

The region associated with the origin of a species is often considered the center of genetic diversity, although this may not be the case with species that have been subject to prolonged periods of domestication and secondary radiation. This has been observed in the common bean (*Phaseolus vulgaris*), where simple sequence repeat (SSR) genetic marker comparisons between native American germplasm and secondary domesticated groupings revealed a higher level of nuclear diversity in the African germplasm than that of the native collection, suggesting that non-native geographical regions can act as both source and sinks for genetic diversity over different historical periods (Bellucci et al., 2014). Palaeobotanical evidence in the form of pollen deposits and historical accounts would strongly suggest domestication of *Cannabis* in the post-Neolithic era over tens of thousands of years, with subsequent secondary domestication events in non-native geographical regions (Li, 1974; Bradshaw et al., 1981; Small and Marcus, 2002; Murphy et al., 2011; Herbig and Sirocko, 2013; Duvall, 2016).

Based on field observations it has been concluded that *Cannabis* originated within Central Asia (Hillig, 2005b; Russo, 2007) and references therein, although such inferences may not be justified given the domestication and radiation of *Cannabis* throughout Eurasia over several millennia (Li, 1974; Bradshaw et al., 1981; Murphy et al., 2011; Herbig and Sirocko, 2013). Conclusive phylogenetic evidence in support of a specific geographical region is also incomplete, with ruderal or wild populations underrepresented or absent from sample collections (Hillig, 2005b; Lynch et al., 2015). Combining genetic and phenotypic evidence relating to the predominant characteristics of a species in both ruderal and domesticated forms in the context of their allelic eco-geographical distribution, is more likely to define a putative center of diversity, as has been noted in other widely cultivated plant species (Smith and King, 2000).

East Asia appears to be a rich source of genetic diversity within the *Cannabis* genepool, and a potentially valuable genetic resource both for future phylogenetic analyses and *ex situ* conservation. China is botanically megadiverse (Li, 2008). The Hengduan Mountains in the south west of China have been identified as one of only 35 biodiversity hotspots worldwide (Sloan et al., 2014) and this region encompasses parts of the Yunnan province in which the Yunnan Academy of Agricultural Sciences (YAAS) *Cannabis* germplasm collection is maintained. China also benefits from a latitudinal gradient from ~23–50°N (Amaducci et al., 2014), with hundreds of *Cannabis* landraces reported to have undergone distinct domestication events along these latitudes in provinces spanning from Hebei in the north west, through to ShanDong, Henan, Guizhou and Yunnan in the south west (Salentijn et al., 2014). Moreover, historical evidence strongly suggests that *Cannabis* has been cultivated in China over several thousand years. Pottery paintings depicting *Cannabis* are believed to have been produced by the Neolithic Yangshao culture, and *Cannabis* fibers were reportedly utilized in the production of paper during the Han dynasty >1790 years before present (Li, 1974). Excavation of the 2700 year old Yanghai Tombs in Xinjiang-Uyghur Autonomous Region in China has also revealed high THC plant material (Russo et al., 2008), implying that this plant was used within a cultural and potentially medicinal context within early Chinese societies.

A number of genetic markers have been used to determine levels of heterozygosity in Chinese *Cannabis* germplasm (Table 1), with SSR, amplified fragment length polymorphism (AFLP) and randomly amplified polymorphic DNA (RAPD) genetic markers indicating a high level of genetic diversity, with the proportion of polymorphic loci ranging from 75 to 92% (Gilmore et al., 2007; Hu et al., 2012; Gao et al., 2014; Zhang et al., 2014). Analysis of 76 accessions from 26 countries using 12 chloroplast and mitochondrial DNA loci revealed six haplotypes, all of which were located within or adjacent to China (Gilmore et al., 2007). Fifty six loci derived from expressed sequence tag (EST) simple sequence repeat (EST-SSR) markers were tested on a sample collection of 100 varieties from 10 provinces in China and 15 varieties from Europe. Principle coordinate analysis revealed four clusters relating to geographical location and latitude. Interestingly, clusters from Central, Northern, and Southern China had a higher percentage of polymorphic loci than the European cluster (Gao et al., 2014; Table 1), suggesting a higher level of diversity within Chinese germplasm.

**Table 1:** **Evidence for the diversity of East Asian germplasm**.

**DNA marker**	**No. loci /bands**	**No. of accessions**	**Country of origin of accessions**	**Province of Chinese accessions**	**Genetic variability**	**References**
AFLP®	442	49	Europe, China	Anhui, Gansu, Guangxi, Guizhou, Heilongjiang, Inner Mongolia, Jilin, Kunming, Liaoning, Shanxi, Sichuan, Xinjiang, Yunnan	Percentage of polymorphic loci (PPL): 92.1%	Hu et al., 2012
					Yunnan (88.8%) and Heilongjiang (75.6%) populations exhibited the highest PPL	
CpSSR / mtSSR	7	76	Afghanistan, Australia, Canada, China, Former Czechoslavakia, Former East Germany, Former USSR, France, Hungary, India, Italy, Jamaica, Japan, Korea, Lebanon, Nepal, Netherlands, Romania, Sierra Leone, South Africa, Swaziland, Thailand, Turkey, Uganda, USA, Zimbabwe	Not specified	Six organelle haplotypes identified	Gilmore et al., 2007
					All haplotypes occurred in South and East Asia, either within China or countries adjacent to China	
EST-SSR	56	115	China, France, Poland, Ukraine	Anhui, Chongqing, Gansu, Gansu, Hanma, Hebei, Heilongjiang, Henan, Huangzhangma, Jinlin, Liaoning, Neimenggu, Ningxia, Shandong, Shanxi, Xinjiang, Yunnan, Zhejiang	PCoA: accessions clustered into four groups; Central, Northern and Southern China, and Europe	Gao et al., 2014
					Germplasm originating from Central China (85.5%), Northern China (76.4%), and Southern China (74.6%) exhibited the highest PPL compared with germplasm originating from Europe (41.8%)	
ISSR and chromosome	183	27	China	Anhui, Chongqing, Hebei, Heilongjiang, Henan, Inner Mongolia, Jiangsu, Jilin, Liaoning, Ningxia, Qianghai, Shananxi, Shandong, Shanxi, Xinjiang, Yunnan, Zhejiang	PPL: 85.8%	Zhang et al., 2014
					Five chromosome types 2*n* = 20 = 14m + 6sm; 2*n* = 20 = 20m; 2*n* = 20 = 18m + 2sm; 2*n* = 20 = 16m + 4sm; and 2*n* = 20 = 12m + 8sm	
					3 karyotypes identified	
RAPD	106	16	China	Anhui, Heilongjiang, Inner Mongolia, Jilin, Liaoning, Shandong, Tibet, Yunnan, Xinjiang	PPL: 74.5%	Tang et al., 2013

**Figure 3:**
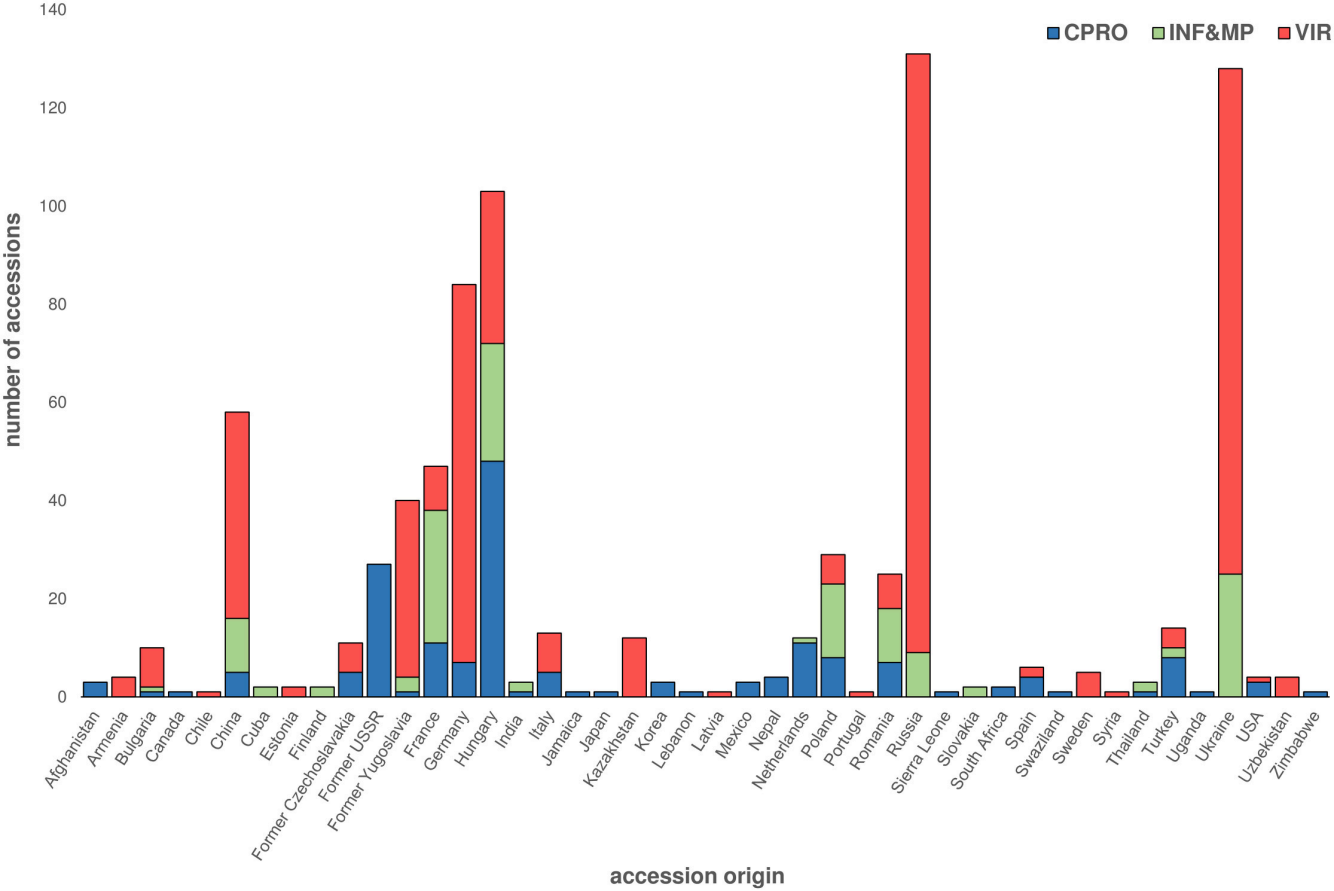
**Summary of CPRO, INF&MP, and VIR published ***Cannabis*** germplasm resources**. Number and associated country of origin of accessions listed in CPRO, INF&MP, and VIR germplasms. CPRO accessions listed in De Meijer and van Soest (1992) and Gilmore et al. (2007); INF&MP listed in Mankowska and Silska (2015); VIR accessions listed in Clarke (1998); Center for Plant Breeding and Reproduction Research, CPRO; Institute of Natural Fibers and Medicinal Plants, INF&MP; Vavilov Research Institute gene bank, VIR.

Of the 808 accessions reported to have been collected within *ex situ Cannabis* genetic resource collections worldwide, only 58 (7.2%) have origins within China (Figure 3). Moreover, from the 156 accessions listed in the former Centre for Plant Breeding and Reproduction Research (CPRO) germplasm collection, cultivars of predominantly European origin and marijuana varieties contributed to more than 40% of all accessions (De Meijer and van Soest, 1992). Considering the long cultivation history of *Cannabis* in China, and the high density of landrace accessions occurring within a large latitudinal range, sourcing accessions from China and adjacent regions should be a priority for *Cannabis ex situ* conservation, irrespective of whether the center of origin for this species has been fully characterized. Nevertheless, appropriate management of germplasm *ex situ* and systematic characterization of East Asian *Cannabis* genetic resources will be required if the full potential of genepool-enabled crop improvement is to be maximized.

## Limited coordination of *Cannabis* genetic resources

Limited effort has been devoted to comprehensive characterization of currently available *Cannabis ex situ* germplasm resources. In the latter part of the twentieth century *Cannabis* genetic resource collections in North America were confiscated, or in some instances reported to have been destroyed by regulatory authorities (Small and Marcus, 2002). Those that did remain were subject to funding constraints, resulting in suboptimal maintenance, and regeneration of seed (Clarke, 1998). However, an increasing number of research groups are currently active in *Cannabis* research (see Supplementary Table), and a concerted effort to characterize *ex situ* genetic resource collections would have significant benefit to the research community and for the continued genetic improvement of this plant.

*Ex situ Cannabis* genetic resource collections have been partially characterized and reported within peer reviewed conference proceedings and other reports from public sector researchers, as well as in a variety of gray literature and online databases (Table 2). These include reference to collections in Europe from the Research Institute of Crop Production (RICIP; Pavelek, 2006), the Leibniz Institute of Plant Genetics and Crop Plant Research (IPK; Höppner, 2006), and the Istituto Sperimentale per le Colture Industriali (CRA-ISCI; Mandolino et al., 2006). The characterization of three genetic resource collections has been published in refereed scientific journals; the CPRO germplasm collection (De Meijer and van Soest, 1992), the Vavilov Research Institute (VIR; Clarke, 1998) and the Institute of Natural Fibres and Medicinal Plants (INF&MP) gene bank collections (Mankowska and Silska, 2015).

**Table 2:** **Summary of ***Cannabis*** germplasm resources reported within the literature**.

***Cannabis* genetic resource collection**	**Location**	**Link**	**Online database**	**References**
Centre for Plant Breeding and Reproduction Research (CPRO)	Netherlands-Wageningen	http://www.wageningenur.nl/	n/a	De Meijer and van Soest, 1992; De Meijer et al., 1992; Hillig, 2005b; Gilmore et al., 2007
Ecofibre Global Germplasm Collection	Australia, Queensland	http://www.ecofibre.com.au/	n/a	Welling et al., 2015
HortaPharm B.V., Amsterdam, Holland (now merged with GW pharmaceuticals)	Netherlands, Amsterdam	n/a	n/a	Ranalli, 2004; Hillig, 2005b; Datwyler and Weiblen, 2006
Institute of Natural Fibers and Medicinal Plants (INF&MP)	Poland-Poznan	http://archive-ecpgr.cgiar.org/fileadmin/www.ecpgr.cgiar.org/Presentations/Fibre_crops_NL_2006/Collection%20of%20cannabis%20at%20INF.pdf	http://archive-ecpgr.cgiar.org/	Mankowska and Silska, 2015
Istituto Sperimentale per le Colture Industriali (CRA-ISCI)	Italy, Bologna	http://archive-ecpgr.cgiar.org/fileadmin/www.ecpgr.cgiar.org/Presentations/Fibre_crops_NL_2006/Italy%20Country%20report.pdf	http://archive-ecpgr.cgiar.org/	Mandolino et al., 1999, 2006; Pacifico et al., 2006
Leibniz Institute of Plant Genetics and Crop Plant Research (IPK)	Germany, Gatersleben	http://archive-ecpgr.cgiar.org/fileadmin/www.ecpgr.cgiar.org/Presentations/Fibre_crops_NL_2006/Germany%20Country%20report%20final.pdf	http://gbis.ipk-gatersleben.de/gbis_i/	Small and Marcus, 2003; Höppner, 2006
National Cannabis Collection (NCC)	Hungary, Tápiószele	http://archive-ecpgr.cgiar.org/fileadmin/www.ecpgr.cgiar.org/Presentations/Fibre_crops_NL_2006/Hungary%20Country%20report.pdf	http://archive-ecpgr.cgiar.org/	Simon, 2006
Research Institute of Crop Production (RICIP; owned by AGRITEC Ltd)	Prague, Ruzyne	http://archive-ecpgr.cgiar.org/fileadmin/www.ecpgr.cgiar.org/Presentations/Fibre_crops_NL_2006/Czech%20Country%20report.pdf	http://archive-ecpgr.cgiar.org/	Small and Marcus, 2003; Pavelek, 2006
Vavilov Research Institute (VIR)	Russia-St Petersburg	http://www.vir.nw.ru/	http://91.151.189.38/virdb/	Clarke, 1998; Small and Marcus, 2003; Hillig, 2005b
Yunnan Academy of Agricultural Sciences (YAAS)	China, Kunming	http://www.university-directory.eu/China/Yunnan-Academy-of-Agricultural-Sciences.html	n/a	HuaDong and YingFang, 2012; Salentijn et al., 2014

*It should be noted that the availability of Cannabis germplasm may vary over time between genetic resource collections and legislatures*.

The CPRO (now the Wageningen University) germplasm collection was established in 1988 (De Meijer and van Soest, 1992; Table 2). This is the only published example of a collection which provides passport data for each accession (De Meijer and van Soest, 1992; Clarke, 1998; Mankowska and Silska, 2015). The last comprehensive report in 1992 identified over 156 accessions (De Meijer and van Soest, 1992; Gilmore et al., 2007), comprising of cultivars, varieties, strains or forms, as defined by International Code of Nomenclature for Cultivated Plants (Brickell et al., 2009), as well as landrace, feral, or ruderal accessions. Accessions were sourced from previous genetic resource collections and academic and commercial organizations. Accessions held within this collection originated from more than 22 countries (De Meijer and van Soest, 1992; Gilmore et al., 2007) (Figure 3), with 42.6% originating from the former Union of Soviet Socialist Republics (USSR) and Hungary. The last published entry of a CPRO accession was in 1999 (Gilmore et al., 2007), although this collection is now being used in the EU-led MultiHemp consortium (Salentijn et al., 2014).

The VIR collection is considered the largest collection of *Cannabis* genetic resources in Europe (Small and Marcus, 2002; Table 2). The VIR, formerly the All-Union Institute of Plant Industry, established in 1920 by Nikolai Vavilov was the first *ex situ* gene bank of its kind to exist worldwide (Tyagi and Agrawal, 2015). The last published report in 1998 characterized the origin of 493 accessions, with accessions from Ukraine, Russia, and Germany representing 61.3% of the collection (Figure 3; Clarke, 1998). Lack of funding is thought to have significantly compromised regeneration efforts (Khoury et al., 2010) leading to a loss of unique genetic diversity held within this resource (Clarke, 1998; Small and Marcus, 2002).

The INF&MP gene bank is the most recently published genetic resource collection (Mankowska and Silska, 2015; Table 2). A report in 2015 identified 139 accessions of predominantly European Origin, with accessions from France, Hungary, and the Ukraine contributing to 54.7% of the collection (Mankowska and Silska, 2015; Figure 3). However, passport data of individual accessions held within the INF&MP and VIR collections are not listed within the literature, with both articles limiting data to the number of accessions, and their associated country of origin (Clarke, 1998; Mankowska and Silska, 2015). Further, provenance is unclear, since the authors do not clearly define the term origin, which can be interpreted as either the location that the plant exists within its natural state, the location with which the accession was acquired, or in the case of a cultivar, strain, variety or form, the location where the accession was developed or registered.

Reference to other significant genetic resource collections has also been made in the literature, including the YAAS collection in China-Yunnan, comprising of ~350 accessions of mainly East Asian origin (Salentijn et al., 2014), and the Ecofibre Global Germplasm Collection (EFGGC) in Australia which has a diverse collection of accessions from multiple regions within Eurasia (Welling et al., 2015). However, comprehensive published data detailing the extent of genetic resources held within these collections is lacking. In the absence of more contemporary peer reviewed published data that systematically characterizes genetic resources, along with detailed passport and other metadata, estimates of the extent to which genetic resource collections represent the global allelic diversity of *Cannabis* will be incomplete.

## Addressing the fragility of global *Cannabis* germplasm resources

### Maintenance and regeneration

Regeneration is one of the most costly factors in the conservation of *ex situ* genetic resource collections and requires significant input in the form of labor, resources, and infrastructure (Li and Pritchard, 2009; Khoury et al., 2010). The regeneration of *Cannabis* germplasm is especially problematic as plants are anemophilous (wind pollinated) and dioecious, with male or hermaphrodite plants releasing large amounts of pollen during flowering (Amaducci et al., 2014). It has been estimated that a single plant can produce in excess of 350 million pollen grains (Faegri et al., 1989). Prevention of gene flow and hybridization between accessions is therefore an important consideration in *Cannabis ex situ* regeneration and conservation (De Meijer and van Soest, 1992), and it may have contributed to reports of a widely shared *Cannabis* genepool.

Outdoor regeneration can require large areas of land, with distances of up to 5 km required to prevent cross-pollination (De Meijer and van Soest, 1992; Small and Antle, 2003). However, given the long distances *Cannabis* pollen can travel and the sensitivity of pollen distribution to wind velocity (Small and Antle, 2003), as well as the potential for prolonged viability of *Cannabis* pollen in low relative humidities post-anthesis (Bassani et al., 1994), introgression may not necessarily be prevented at these distances. Outdoor regeneration can also be impractical where multiplication of diverse accessions requires specific photoperiods spanning several degrees of latitude (Gao et al., 2014) in order to initiate flowering (Cosentino et al., 2012), thus limiting multiplication of *Cannabis* germplasm to certain periods throughout the year.

Protected cultivation within pollen secure facilities is a standard alternative to outdoor multiplication. However, these may be costly and also problematic in maintaining genetic diversity. For many plant species, genetic drift has been attributed to the process of regeneration, and associations have been detected between time spent *ex situ* and loss of alleles per locus, gene diversity, and percentage of polymorphic loci (Parzies et al., 2000). For example, loss of *ex situ* genetic diversity has been observed in outcrossing species such as barley (*Hordeum vulgare* L.), maize and common bean genetic resource collections (Parzies et al., 2000; Rice et al., 2006; Negri and Tiranti, 2010), although much of the reduction in genetic diversity appears dependent on the number of parents used in mass pollination (Parzies et al., 2000). Given the spatial limitations associated with the construction of indoor pollen-secure infrastructure (Negri and Tiranti, 2010), which limits the number of outbreeding individuals for regeneration, a decline in allelic variation may occur with each regeneration cycle, ultimately leading to erosion of genetic variability from the time of acquisition.

### Seed storage

Considering the complexities associated with *Cannabis* germplasm regeneration, much attention has been directed to efficient methods of seed storage. Duration of storage, temperature, and seed moisture content are all variables which can significantly affect the viability of *Cannabis* seeds (Small and Brookes, 2012; Parihar et al., 2014). Common practice for short-term seed storage is 4°C with a moisture content of 6% (De Meijer and van Soest, 1992; Small and Brookes, 2012; Mankowska and Silska, 2015), while for longer periods of storage >3 years, seeds are held at −20°C with ~4% moisture content (De Meijer and van Soest, 1992; Mankowska and Silska, 2015). *Cannabis* seed appears to have orthodox storage behavior, and the ability to withstand periods of up to 66 months after desiccation with minimal effects on seed viability (Small and Brookes, 2012; Parihar et al., 2014). Nevertheless, systematic evidence for the long-term viability of *Cannabis* seed is lacking. Given the variation in seed size amongst *Cannabis* germplasm (Piluzza et al., 2013), it is important to establish optimal periods for seed storage prior to multiplication.

Desiccation and cold storage does not necessarily guarantee seed longevity: Only 61/276 species were found to exhibit a half-life >100 years at these conditions (Walters et al., 2005). Both orthodox and non-orthodox seed types may benefit from cryopreservation conditions (Li and Pritchard, 2009; Michalak et al., 2015; Perullo et al., 2015; Prada et al., 2015). Despite the absence of data supporting the long-term viability of seed storage at temperatures below −180°C, cryopreservation of seed has successfully been demonstrated as a proof of concept in a number of plant species (Li and Pritchard, 2009; Michalak et al., 2015; Perullo et al., 2015; Prada et al., 2015). Short-term cryopreservation of seed of several species, including black poplar (*Populus nigra* L.; Michalak et al., 2015), swamp pink (*Helonias bullata* L.; Perullo et al., 2015), and Barbados nut (*Jatropha curcas* L.; Prada et al., 2015), had no detrimental effect on germination. However, seed water content prior to immersion in liquid nitrogen can significantly determine seed viability (Michalak et al., 2015). Given the expense and propensity for genetic drift associated with *Cannabis* germplasm regeneration, as well as the potential orthodox storage properties of *Cannabis* seed, contemporary methodologies of seed storage should be explored to determine if they are compatible and economically feasible with long-term *Cannabis ex situ* conservation.

Few studies have attempted to monitor seed aging in *Cannabis* (Parihar et al., 2014) or associate a specific biochemical, metabolic, or physiological characteristic with seed viability or vigor (Small and Brookes, 2012). However, a number of novel biomarker-based prediction tools have been developed, with the potential to monitor seed aging beyond traditional germination and biochemical tests (Fu et al., 2015). Biomarkers associated with DNA methylation (Rocha et al., 2005) as well as FA (Li et al., 2007) and endogenous antioxidant metabolism (Revilla et al., 2009) have been found to be reliable at predicting seed aging (Fu et al., 2015). Such tools may contribute toward predicting *Cannabis* seed viability, and be adopted for development of “best practice” methods for long-term *ex situ* seed management.

### Passport data

The long-term value and utility of *ex situ* germplasm is subject to the quality and quantity of data and metadata available for a given genetic resource (Ramírez-Villegas et al., 2010; Endresen et al., 2011). The systematic collation of passport data can aid in identifying duplication which may have occurred both between and within genetic resource collections (Van Hintum and Visser, 1995) Indeed, the recently revised gene bank standards for plant genetic resources recommend the comprehensive assignment of universally accepted passport descriptors, and that the conservation and use of genetic resources should be collated within a suitably designed database (Tyagi and Agrawal, 2015). The adoption of Digital Object Identifiers (DOIs) for datasets and associated entities, linked to an Open Researcher and Contributor ID (ORCID) can also encourage data sharing and reduce management complexities associated with homonyms and synonyms.

Gap methodologies which incorporate sampling representativeness scores and distribution modeling can be utilized to prioritize geographical locations for future *ex situ* conservation. These methods have been successfully applied to intra- and inter-specific taxa of the common bean (Ramírez-Villegas et al., 2010), Pigeon pea (*Cajanus cajan* L.), African cowpea (*Vigna unguiculata* L.; Moray et al., 2014), wheat, barley (Endresen et al., 2011), and tomato (Castañeda-Álvarez et al., 2015). Determining the eco-geographical profile or biome-specific adaption of germplasm with the use of passport data, geographic information system (GIS) technologies (http://www.diva-gis.org), and modeling software (Endresen, 2010; Ramírez-Villegas et al., 2010) can also increase the probability of targeting phenotypically relevant accessions within *ex situ* genetic resource collections (Bekessy et al., 2003; Endresen, 2010; Endresen et al., 2011; Jones et al., 2013). However, in the case of *Cannabis* genetic resources, where comprehensive publicly available passport, provenance, and metadata are either absent or deemed low-quality, these approaches to *Cannabis* germplasm characterization and conservation are currently not possible.

## A novel approach to *Cannabis* conservation

### Establishing a virtual core collection

Additional approaches have been developed to enhance the management of *ex situ* seed genetic resource collections. These include the establishment of core collections, which represent the range of genetic diversity for a species with minimal repetition (Brown, 1989). Whilst core collections contain a subset of the genetic resources for a species, they do provide an enriched source of diversity, enabling efficient genepool characterization and utilization (Brown, 1989; Odong et al., 2013). We propose that an international core collection of *Cannabis* be established from existing gene banks comprising up to 400 accessions collected from diverse geographical regions. Based on theoretical studies of natural populations (Lawrence et al., 1995a), in *ex situ* collections this should represent 99% of allelic polymorphisms for alleles that occur at species-wide frequencies >2% (Lawrence et al., 1995b). Having established an initial core collection, more comprehensive empirical testing could employ methods which use pairwise comparisons of allele frequencies of the core to guide the inclusion or exclusion of additional accessions (Thachuk et al., 2009; Odong et al., 2013).

Major barriers to the immediate development of a centralized physical core collection for *Cannabis*, include the lack of publicly accessible seed banks (Sawler et al., 2015) and the limitations on import, export, and transfer of seed between sovereign states that are signatories to the United Nations 1961 Single Convention on Narcotic Drugs and the 1971 Convention on Psychotropic Substances (Nutt, 2015; Pain, 2015). Nevertheless, this need not prevent the generation and exchange of data, nor the development of internationally agreed nomenclature and characterization standards, as well as the establishment of a “virtual” core collection and associated online data repository. Indeed, considering these obstacles, the *in silico* management of genetic resources and development of a commonly shared database containing detailed passport, meta-, and characterization-data, together with a coordinated distribution system, may be a necessity for *ex situ Cannabis* conservation, and even facilitate the commodification of privately managed germplasm collections as a pre-breeding resource.

### High throughput DNA sequencing

Industrial hemp displays considerable phenotypic plasticity in response to environmental factors (Struik et al., 2000; Amaducci et al., 2014). Thus, characterization of genetic diversity based on morphological characteristics (Jansky et al., 2015) or eco-geographical data alone (Jones et al., 2013) are not always accurate indicators of diversity within genetic resource collections. Neutral DNA markers, such as SSR, RAPD, and AFLP, which may not be subject to selective pressures, have typically been employed to assess the level of genetic diversity and phylogeny between *Cannabis* genetic resources (Mandolino and Carboni, 2004; Table 1). However, these do not necessarily reflect quantitative patterns of trait variation, nor can they always be considered a reliable tool in characterizing the functional genetic diversity of a species, nor the extent of genetic structure required for conservation (Bekessy et al., 2003).

High density SNP genotyping offers the promise of characterizing the genomic architecture underlying plant phenotypic variation and the extent of allelic diversification held within germplasm collections (Kilian and Graner, 2012; Scossa et al., 2015; Varshney et al., 2015). This has been demonstrated in other species (Kim and Buell, 2015; Luo, 2015), such as pepper (*Capsicum annuum*; Devran et al., 2015), lupin (*Lupinus angustifolius* L.; Yang et al., 2012), soybean (Kadam et al., 2016), and tomato (Kaminski et al., 2016). High-throughput multi-parallel resequencing would also promote sequence informed *ex situ* conservation and could potentially eliminate germplasm redundancy (Kilian and Graner, 2012), and may further aid in resolving the plasticity of *Cannabis* traits which appear associated with epigenetic regulation, such as sex differentiation (Razumova et al., 2015).

Using high-throughput DNA sequencing to characterize *Cannabis* genome diversity and to develop markers is currently limited, due to the lack of a chromosome anchored and fully annotated reference genome, which limits sequencing applications (Ekblom and Galindo, 2011; Scossa et al., 2015). Two draft genomes are available for *Cannabis* (http://www.medicinalgenomics.com; http://genome.ccbr.utoronto.ca/; Van Bakel et al., 2011) and these have been characterized through the completion of >195 K scaffolds. *De novo* sequencing is now widely utilized in non-model species, although this approach has a number of biological, computational, and biomolecular challenges specific to plants; (Schatz et al., 2012), such as the relatively high levels of heterozygosity (Gore et al., 2009) and genome complexity (Schnable et al., 2009). Reduced genome representation and complexity sequencing can also be utilized in the absence of a contiguous reference genome (Davey et al., 2011; Kim et al., 2016), although library preparation and choice of restriction enzyme can result in erroneous genotyping due to method induced sequencing errors, low read depth (Kim et al., 2016), uneven marker density and ultimately, missing data (Beissinger et al., 2013; Gardner et al., 2014).

### Data integration

The accumulation of high-throughput DNA sequence data requires systematic management of large datasets (Kilian and Graner, 2012) and analysis to obtain meaningful assemblies (Lange, 2016) that correspond to causative genomic regions and the underlying phenotypic traits of interest (Love et al., 2012). Navigation between data from different realms of genomics, genetics and trait-phenomics also requires a detailed understanding of the underlying genetic and experimental resources. The collation of data, however, can be incorporated into a suite of interconnected online databases such as those outlined for InterStoreDB (www.interstoredb.org/), which provides a web interface between raw sequence data, pairwise alignments and metadata relating to provenance, phenotype, and experimental parameters (Love et al., 2012). This framework incorporates the generic CropStoreDB (http://www.cropstoredb.org) database, which is currently being developed by researchers at Southern Cross University in Australia to characterize candidate genes involved in cannabinoid and lignocellulose composition in *Cannabis*.

*Cannabis* genome and transcriptome data are currently scattered within a number of public depositories (see review Lange, 2016), including the National Centre for Biotechnology Information (NCBI) website (http://www.ncbi.nlm.nih.gov/; Van Bakel et al., 2011; Lynch et al., 2015; Sawler et al., 2015). Phenotype-specific data have also been made available through databases such as TrichOME (Dai et al., 2010; Lange, 2016), which incorporates ESTs and metabolite data from the trichomes of various aromatic plant species including *Cannabis* (Dai et al., 2010), although no entries post 2012 have been recorded (Lange, 2016). A coordinated effort by researchers to centralize and normalize these data sets has the potential not only to harbor collaborative analysis, but also accelerate the *in silico* discovery of complex gene-to-trait relationships, ultimately benefiting both the research community and plant breeders alike.

## Conclusion

*Cannabis* is a phenotypically and genetically diverse genus which has yet to benefit from the advanced level of breeding applied to other commercial crops. Coordinated and comprehensive conservation and characterization of *ex situ Cannabis* resources holds the promise of preserving genepool diversity and enabling cultivar development. However, the legal constraints imposed by international narcotics conventions over more than 50 years have been influential in the fractionation and erosion of publicly accessible *Cannabis ex situ* genetic resources. The restrictions on legal exchange of *bona fide* research materials continues to limit the establishment of physical and centralized *ex situ* core collections. Nevertheless, the advent of low cost high-throughput DNA sequencing technologies and user-friendly data and metadata online repositories, make it possible to develop a virtual *Cannabis* core collection to facilitate the *in silico* mining of trait diversity and to guide genetic improvement strategies. This approach not only has the potential to accelerate the introgression and stabilization of commercially valuable genes into desirable germplasm, but may also precipitate a Green Revolution for *Cannabis* and the renewed commercial exploitation of this multi-functional plant species.

## Author contributions

MW: Carried out detailed literature survey and prepared manuscript. TS: Contributed to literature review and background information. TR: Contributed to development of review topic, detailed review, and revision of manuscript. LL: Contributed to organization of manuscripts, along with review and revision. RS: Contributed to generation of image data and review of manuscript. GK: Conceived of review topic, detailed review, and revision of manuscript.

## Funding

MW, TS, and RS were sponsored by scholarships funded by Ecofibre Industries Operations Pty Ltd. TS is an employee of Ecofibre Industries Operations Pty Ltd.

### Conflict of interest statement

The authors declare that the research was conducted in the absence of any commercial or financial relationships that could be construed as a potential conflict of interest.
