# Supplementary material for: A Belated Green Revolution for Cannabis: Virtual Genetic Resources to Fast-Track Cultivar Development
Source: Front Plant Sci. 2016 Jul 29;7:1113. doi: 10.3389/fpls.2016.01113 (PMC4965456; doi:10.3389/fpls.2016.01113)
Supplement: Supplementary file 1 [file Table1.DOCX]

Supplementary Material

A Belated Green Revolution for Cannabis: Virtual Genetic Resources to Fast-Track Cultivar Development

Matthew T Welling, Tim Shapter, Terry J Rose, Lei Liu, Rhia Stanger, Graham J King*

*** Correspondence:** graham.king@scu.edu.au

# Supplementary Table

**Supplementary Table. Summary of research groups currently active in *Cannabis* research.** We define active as having published one or more manuscripts in scholarly peer reviewed journals within the last 12 months, where these are identified on the basis of the search terms “*Cannabis*” & “industrial hemp” in Scopus.

| **Research institution/group/organization** | **Reference** |
| --- | --- |
| Agriculture and Agri-Food Canada, Science and Technology Branch, Ontario, Canada | (Small and Naraine, 2015) |
| Beijing University of Chemical Technology, State Key Laboratory of Chemical Resource Engineering, Beijing Key Laboratory of Electrochemical Process and Technology for Materials, China | (Wang et al., 2015) |
| Chinese Academy of Agricultural Science, Institute of Bast Fiber Crops, Changsha, Hunan, China | (Chaohua et al., 2016) |
| Consiglio per la ricerca in agricoltura e l’analisi dell’economia agraria, Centro di Ricerca per le Colture Industriali, Italy | (Onofri et al., 2015) |
| Czech University of Life Sciences, Department of Sustainable Technologies, Prague, Czech Republic | (Kolarikova et al., 2015) |
| Dalhousie University, Department of Plant and Animal Sciences, Faculty of Agriculture, Truro, Nova, Canada | (Sawler et al., 2015) |
| Environmental Research and Innovation, Luxembourg Institute of Science and Technology, Esch-sur-Alzette, Luxembourg | (Andre et al., 2016) |
| French Institute of Science and Technology for Transports, Development and Networks (IFSTTAR), Nantes, France | (Andrianandraina et al., 2015) |
| Institute of Natural Fibres and Medicinal Plants (INF&MP), Poznan, Poland | (Mankowska and Silska, 2015) (Wielgus et al., 2012) |
| Istituto di Agronomia, Genetica e Coltivazioni erbacee, Facoltà di Agraria, Università Cattolica del Sacro Cuore, Piacenza, Italy | (Amaducci et al., 2014) |
| Istituto di Biologia e Biotecnologia Agraria (IBBA-CNR), Milan, Italy | (Galasso and Ponzoni, 2015) |
| McGill University, Department of Plant Science, Québec, Canada | (Vonapartis et al., 2015) |
| Russian State Agrarian University, Center for Molecular Biotechnology, Moscow, Russia | (Razumova et al., 2015) |
| Southern Cross University, Southern Cross Plant Science, New South Wales, Australia | (Welling et al., 2015) |
| Technical University of Denmark, Center for Bioprocess Engineering, Department of Chemical and Biochemical Engineering, Søltofts Plads, Lyngby, Denmark | (Liu et al., 2015) |
| Université Paris Descartes, Faculté des Sciences Pharmaceutiques et Biologiques, France | (Ribeiro et al., 2015) |
| University College London, Department for Pharmaceutical and Biological Chemistry, Centre for Pharmacognosy and Phytotherapy, London, UK | (Peschel and Politi, 2015) |
| University of Alberta, Biorefining Conversions and Fermentations Laboratory, Department of Agricultural, Food and Nutritional Science, Edmonton, Canada | (George et al., 2015) |
| University of Bielsko-Biala, Faculty of Materials, Civil and Environmental Engineering, Bielsko-Biala, Poland | (Kuglarz et al., 2016) |
| University of British Columbia, Botany, Department, Vancouver, BC, Canada | (Sawler et al., 2015) |
| University of Chemical Technology Prague, Department of Carbohydrates and Cereals, Prague, Czech Republic | (Švec and Hrušková, 2015) |
| University of Melbourne, School of Applied Sciences, Victoria, Australia | (Pandohee et al., 2015) |
| University of Minnesota, Department of Plant Biology and Bell Museum, St Paul, USA | (Weiblen et al., 2015) |
| University of Mississippi, National Center for Natural Products Research, Mississippi, USA | (Gul et al., 2015) (Chandra et al., 2015) |
| University of New Mexico, Department of Geography and Environmental Studies, Albuquerque, USA | (Duvall, 2016) |
| University of the Basque Country (UPV/EHU), Analytical Chemistry Department, Barrio Sarriena, Leioa, Spain | (Aizpurua-Olaizola et al., 2016) |
| Upytė Experimental Station, the Lithuanian Research Centre for Agriculture and Forestry, Upytė, Panevėžys distr, Lithuania | (Jankauskienė and Gruzdevienė, 2015) (Jankauskienė et al., 2015) |
| Wageningen University and Research Centre, Wageningen UR Plant Breeding, The Netherlands | (Salentijn et al., 2014) |
| Yunnan University, College of Agricultural Science, Kunming, China | (Cheng et al., 2016) |

# Supplementary references

Aizpurua-Olaizola, O., Soydaner, U., Öztürk, E., Schibano, D., Simsir, Y., Navarro, P., et al. (2016). Evolution of the cannabinoid and terpene content during the growth of *Cannabis sativa* plants from different chemotypes. *J. Nat. Prod.* 79**,** 324–331. doi: 10.1021/acs.jnatprod.5b00949

Amaducci, S., Scordia, D., Liu, F.H., Zhang, Q., Guo, H., Testa, G., et al. (2014). Key cultivation techniques for hemp in Europe and China. *Ind Crops Prod.* 68**,** 2-16. doi: 10.1016/j.indcrop.2014.06.041

Andre, C.M., Hausman, J.-F., and Guerriero, G. (2016). *Cannabis sativa*: the plant of the thousand and one molecules. *Front Plant Sci.* **7**:19. doi: 10.3389/fpls.2016.00019

Andrianandraina, E.,Ventura, A., Senga Kiessé, T., Cazacliu, B., Idir, R., and van der Werf, H.M.G. (2015). Sensitivity analysis of environmental process modeling in a life cycle context: a case study of hemp crop production. *J Ind Ecol.* 19**,** 978-993. doi: 10.1111/jiec.12228

Chandra, S., Lata, H., Mehmedic, Z., Khan, I.A., and ElSohly, M.A. (2015). Light dependence of photosynthesis and water vapor exchange characteristics in different high Δ 9-THC yielding varieties of *Cannabis sativa* L. *J Appl Res Med Aromat Plants* 2**,** 39-47. doi: 10.1016/j.jarmap.2015.03.002

Chaohua, C., Gonggu, Z., Lining, Z., Chunsheng, G., Qing, T., Jianhua, C., et al. (2016). A rapid shoot regeneration protocol from the cotyledons of hemp (*Cannabis sativa* L.). *Ind Crops Prod* 83**,** 61-65. doi: 10.1016/j.indcrop.2015.12.035

Cheng, X., Deng, G., Su, Y., Liu, J.J., Yang, Y., Du, G.H., et al. (2016). Protein mechanisms in response to NaCl-stress of salt-tolerant and salt-sensitive industrial hemp based on iTRAQ technology. *Ind Crops Prod* 83**,** 444–452. doi: doi:10.1016/j.indcrop.2015.12.086

Duvall, C.S. (2016). Drug laws, bioprospecting and the agricultural heritage of *Cannabis* in Africa. *Space and Polity* 20**,** 10-25. doi: 10.1080/13562576.2016.1138674

Galasso, I., and Ponzoni, E. (2015). *In silico* exploration of *Cannabis sativa* L. genome for simple sequence repeats (SSRs). *Am J Plant Sci* 6**,** 3244-3250 doi: 10.4236/ajps.2015.619315

George, M., Mussone, P.G., and Bressler, D.C. (2015). Improving the accessibility of hemp fibres using caustic to swell the macrostructure for enzymatic enhancement. *Ind Crops Prod* 67**,** 74-80. doi: [10.1016/j.indcrop.2014.10.043](http://dx.doi.org/10.1016/j.indcrop.2014.10.043)

Gul, W., Gul, S.W., Radwan, M.M., Wanas, A.S., Khan, I.I., Sharaf, M.H., et al. (2015). Determination of 11 cannabinoids in biomass and extracts of different varieties of *Cannabis* using high-performance liquid chromatography. *J AOAC Int* 98**,** 1523-1528. doi: 10.5740/jaoacint.15-095

Jankauskienė, Z., Butkutė, B., Gruzdevienė, E., Cesevičienė, J., and Fernando, A.L. (2015). Chemical composition and physical properties of dew-and water-retted hemp fibers. *Ind Crops Prod* 75**,** 206-211. doi: 10.1016/j.indcrop.2015.06.044

Jankauskienė, Z., and Gruzdevienė, E. (2015). Screening of industrial hemp (*Cannabis sativa* L.) cultivars for biomass yielding capacities in Lithuania. *Journal of Natural Fibers* 12**,** 368-377. doi: 10.1080/15440478.2014.929556

Kolarikova, M., Ivanova, T., Hutla, P., and Havrland, B. (2015). Economic evaluation of hemp (*Cannabis sativa*) grown for energy purposes (briquettes) in the Czech Republic. *Agronomy Research* 13**,** 328-336.

Kuglarz, M., Alvarado-Morales, M., Karakashev, D., and Angelidaki, I. (2016). Integrated production of cellulosic bioethanol and succinic acid from industrial hemp in a biorefinery concept. *Bioresour. Technol.* 200**,** 639-647. doi: 10.1016/j.biortech.2015.10.081

Liu, M., Fernando, D., Daniel, G., Madsen, B., Meyer, A.S., Ale, M.T., et al. (2015). Effect of harvest time and field retting duration on the chemical composition, morphology and mechanical properties of hemp fibers. *Ind Crops Prod* 69**,** 29-39. doi: 10.1016/j.indcrop.2015.02.010

Mankowska, G., and Silska, G. (2015). Genetic resources of *Cannabis sativa* L. in the collection of the gene bank at INF&MP in Poznan. *Journal of Natural Fibers* 12**,** 332-340. doi: 10.1080/15440478.2014.928246

Onofri, C., de Meijer, E.P.M., and Mandolino, G. (2015). Sequence heterogeneity of cannabidiolic- and tetrahydrocannabinolic acid-synthase in *Cannabis sativa* L. and its relationship with chemical phenotype. *Phytochemistry* 116**,** 57-68. doi: 10.1016/j.phytochem.2015.03.006

Pandohee, J., Holland, B.J., Li, B., Tsuzuki, T., Stevenson, P.G., Barnett, N.W., et al. (2015). Screening of cannabinoids in industrial‐grade hemp using two‐dimensional liquid chromatography coupled with acidic potassium permanganate chemiluminescence detection. *J Sep Sci* 38**,** 2024-2032. doi: 10.1002/jssc.201500088

Peschel, W., and Politi, M. (2015). 1 H NMR and HPLC/DAD for *Cannabis sativa* L. chemotype distinction, extract profiling and specification. *Talanta* 140**,** 150-165. doi: 10.1016/j.talanta.2015.02.040

Razumova, O.V., Alexandrov, O.S., Divashuk, M.G., Sukhorada, T.I., and Karlov, G.I. (2015). Molecular cytogenetic analysis of monoecious hemp (*Cannabis sativa* L.) cultivars reveals its karyotype variations and sex chromosomes constitution. *Protoplasma* 253**,** 895-901 doi: 10.1007/s00709-015-0851-0

Ribeiro, A., Pochart, P., Day, A., Mennuni, S., Bono, P., Baret, J.-L., et al. (2015). Microbial diversity observed during hemp retting. *Appl. Microbiol. Biotechnol.* 99**,** 4471-4484. doi: 10.1007/s00253-014-6356-5

Salentijn, E.M.J., Zhang, Q., Amaducci, S., Yang, M., and Trindade, L.M. (2014). New developments in fiber hemp (*Cannabis sativa* L.) breeding. *Ind Crops Prod.* 68**,** 32–41. doi: 10.1016/j.indcrop.2014.08.011

Sawler, J., Stout, J.M., Gardner, K.M., Hudson, D., Vidmar, J., Butler, L., et al. (2015). The genetic structure of marijuana and hemp. *PloS ONE* 10**,** e0133292. doi: 10.1371/journal.pone.0133292

Small, E., and Naraine, S.G. (2015). Size matters: evolution of large drug-secreting resin glands in elite pharmaceutical strains of *Cannabis sativa* (marijuana). *Genet. Resour. Crop Evol.* 63**,** 349-359 doi: 10.1007/s10722-015-0254-2

Švec, I., and Hrušková, M. (2015). Properties and nutritional value of wheat bread enriched by hemp products. *Potravinarstvo* 9**,** 304-308. doi: 10.5219/487

Vonapartis, E., Aubin, M.-P., Seguin, P., Mustafa, A.F., and Charron, J.-B. (2015). Seed composition of ten industrial hemp cultivars approved for production in Canada. *J Food Compost Anal* 39**,** 8-12. doi: 10.1016/j.jfca.2014.11.004

Wang, Y., Yang, R., Li, M., and Zhao, Z. (2015). Hydrothermal preparation of highly porous carbon spheres from hemp (*Cannabis sativa* L.) stem hemicellulose for use in energy-related applications. *Ind Crops Prod* 65**,** 216-226. doi: 10.1016/j.indcrop.2014.12.008

Weiblen, G.D., Wenger, J.P., Craft, K.J., ElSohly, M.A., Mehmedic, Z., Treiber, E.L., et al. (2015). Gene duplication and divergence affecting drug content in *Cannabis sativa*. *New Phytol.* 208**,** 1241–1250. doi: 10.1111/nph.13562

Welling, M.T., Liu, L., Shapter, T., Raymond, C.A., and King, G.J. (2015). Characterisation of cannabinoid composition in a diverse *Cannabis sativa* L. germplasm collection. *Euphytica* 208**,** 463-475. doi: 10.1007/s10681-015-1585-y

Wielgus, K., Przewozna, J., Mankowska, G., Grabowska, L., Podralska, M., and Slomski, R. (2012). Genetic diversity among selected hemp cultivars within the THCA gene. *J. Biotechnol.* 160**,** 264-265. doi: 10.1016/j.jbiotec.2012.06.015
